# RAGE mediated intracellular Aβ uptake contributes to the breakdown of tight junction in retinal pigment epithelium

**DOI:** 10.18632/oncotarget.5894

**Published:** 2015-09-29

**Authors:** Sung Wook Park, Jin Hyoung Kim, Sang Min Park, Minho Moon, Kihwang Lee, Kyu Hyung Park, Woo Jin Park, Jeong Hun Kim

**Affiliations:** ^1^ Fight against Angiogenesis-Related Blindness Laboratory, Biomedical Research Institute, Seoul National University Hospital, Jongno-gu, Seoul, Korea; ^2^ Department of Biomedical Sciences, College of Medicine, Seoul National University, Daehak-ro, Jongno-gu, Seoul, Korea; ^3^ Department of Life Sciences, Life Sciences Concentration GIST (Gwangju Institute of Science and Technology), Cheomdan-gwagiro, Buk-gu, Gwangju, Korea; ^4^ Department of Biochemistry, College of Medicine, Konyang University, Seo-gu, Daejeon, Korea; ^5^ Department of Ophthalmology, Ajou University School of Medicine, Yeongtong-gu, Suwon-si, Gyeonggi-do, Korea; ^6^ Department of Ophthalmology, College of Medicine, Seoul National University, Daehak-ro, Jongno-gu, Seoul, Korea; ^7^ Department of Ophthalmology, Seoul National University Bundang Hospital, Gumiro, Bundang-gu, Seongnam, Gyeonggi-do, Korea

**Keywords:** amyloid β, age-related macular degeneration, endocytosis, tight junction, receptor for advanced glycation end products, Gerotarget

## Abstract

Intracellular amyloid beta (Aβ) has been implicated in neuronal cell death in Alzheimer's disease (AD). Intracellular Aβ also contributes to tight junction breakdown of retinal pigment epithelium (RPE) in age-related macular degeneration (AMD). Although Aβ is predominantly secreted from neuronal cells, the mechanism of Aβ transport into RPE remains to be fully elucidated. In this study, we demonstrated that intracellular Aβ was found concomitantly with the breakdown of tight junction in RPE after subretinal injection of Aβ into the mouse eye. We also presented evidence that receptor for advanced glycation end products (RAGE) contributed to endocytosis of Aβ in RPE. siRNA-mediated knockdown of RAGE prevented intracellular Aβ accumulation as well as subsequent tight junction breakdown in RPE. In addition, we found that RAGE-mediated p38 MAPK signaling contributed to endocytosis of Aβ. Blockade of RAGE/p38 MAPK signaling inhibited Aβ endocytosis, thereby preventing tight junction breakdown in RPE. These results implicate that intracellular Aβ contributes to the breakdown of tight junction in RPE via the RAGE/p38 MAPK-mediated endocytosis. Thus, we suggest that RAGE could be a potential therapeutic target for intracellular Aβ induced outer BRB breakdown in AMD.

## INTRODUCTION

Age-related macular degeneration (AMD) is the leading cause of blindness in the elderly population and the prevalence is increasing in the world [1]. Drusen, a focal deposition of acellular debris between the retinal pigment epithelium (RPE) and Bruch's membrane, is the clinical hallmark and usually the initial clinical finding of AMD. Amyloid β (Aβ) is known to be found in drusen [2, 3]. In addition, the deposition of Aβ in the subRPE space is also concomitant with several features of AMD in a mouse model of Alzheimer disease (AD) [4, 5]. It has been suggested that Aβ in drusen may be an important contributor to the development of AMD.

Drusen in AMD and senile plaque cores in AD are similar in that deposition of Aβ is found in extracellular space at later stages of both diseases. The role of extracellular Aβ in RPE is known to alter the tight junction without apoptosis [6]. Recent studies, on the other hand, suggest that accumulation of intraneuronal Aβ contributes neuronal apoptosis and may be an early event in the pathogenesis of AD [7, 8]. In this regard, we recently reported that intracellular Aβ contributed to the breakdown of outer blood-retinal barrier (BRB) in 5XFAD mice, a mouse model of AD, and suggested that intracellular Aβ could be also a key contributor to the development of AMD [9]. However, the precise mechanism of intracellular uptake of Aβ in RPE remains to be elucidated.

Receptor for advanced glycation end products (RAGE), a member of the immunoglobulin superfamily, is a multi-ligand receptor capable of binding diverse range of molecules including Aβ [10]. The receptor is known to be highly expressed in the RPE and levels of RAGE are significantly elevated in AMD, especially in RPE adjacent to drusen [11, 12]. RAGE is also known to participate in the uptake of Aβ from the circulation into the brain by endocytosis and transcytosis in endothelial cells [13, 14]. Recently, it has been suggested that RAGE-mediated signaling contributes to intraneuronal transport of Aβ [15]. Thus, we hypothesized that RAGE could contribute to intracellular transport of Aβ in RPE. If Aβ uptake is mediated by RAGE in RPE, anti-RAGE therapy could inhibit intracellular accumulation of Aβ, and subsequently prevent the breakdown of tight junction in RPE. This can expand the application of anti-RAGE therapy from AD to AMD [16, 17].

Herein, we demonstrate that subretinal injection of Aβ leads to intracellular uptake of Aβ and subsequent breakdown of tight junction in RPE *in vivo*. We also present evidence that extracellular Aβ gets translocated into intracellular space via RAGE-mediated endocytosis in RPE. The Aβ/RAGE-mediated p38 MAPK signaling contributes to endocytosis of Aβ. Blocking of RAGE inhibits intracellular Aβ-induced tight junction breakdown in RPE. These findings indicate that RAGE contributes to the intracellular transport of Aβ, resulting in increased tight junction breakdown in RPE. Therefore, we suggest that blocking of RAGE can be a potential therapeutic target in AMD.

## RESULTS

### Subretinal injection of Aβ leads to intracellular Aβ uptake and subsequent breakdown of tight junction in RPE

We have recently demonstrated that Aβ, endogenously generated from 5 mutant transgenes, accumulated in intracellular space at the RPE layer with thickened Bruch's membrane and Aβ deposits, thereby attenuating tight junction of RPE in aged 5XFAD mice [9]. Exogenous oligomeric Aβ (OAβ)_42_ injection into subretinal space is also known to break tight junctions of RPE and result in disorganized and irregular staining of both ZO-1 and occludin on days 3 to 15 post-injection in both young and aged mice [6]. These findings led us to probe whether extracellular Aβ translocates into intracellular space and subsequently induces breakdown of tight junction of RPE *in vivo*.

**Figure 1:**
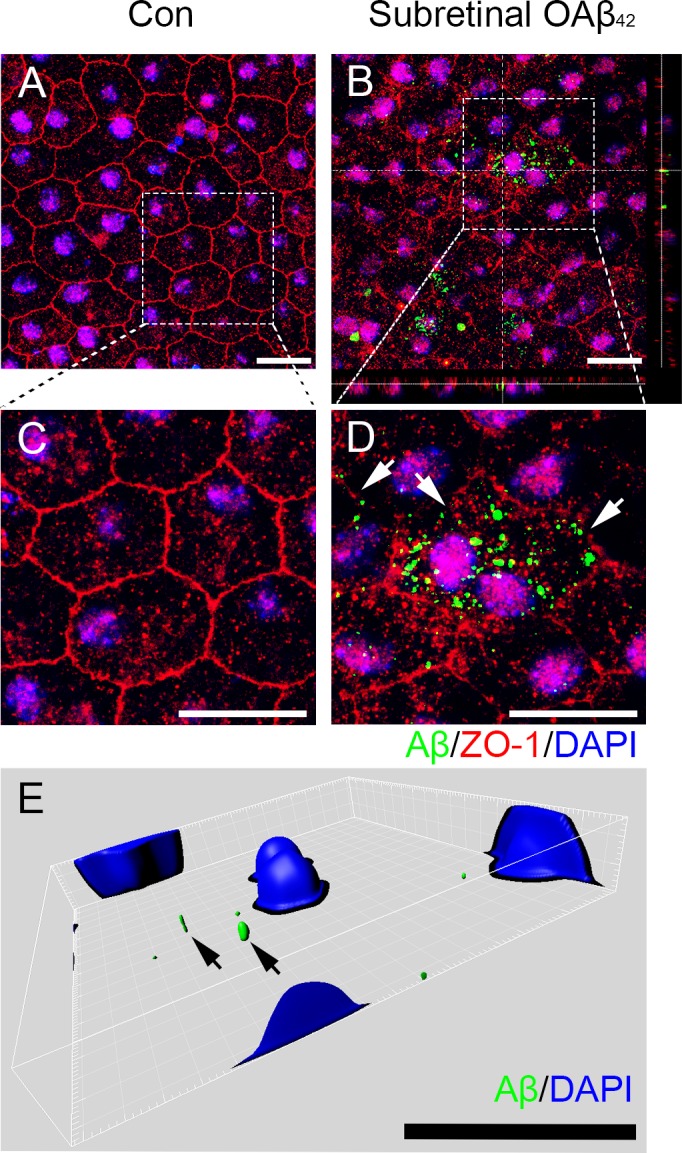
Subretinal injection of Aβ leads to intracellular Aβ uptake and subsequent breakdown of tight junction in RPE Effect of subretinally injected OAβ_42_ (1 μg) on tight junction in retinal pigment epithelium (RPE) flat mount was evaluated at 1 week post injection. **A.**-**E.** RPE flat mounts with immunofluorescence staining against Aβ_42_ (green), tight junction protein ZO-1 (red) and nucleus (DAPI, blue) are shown. **A.** RPE flat mount after subretinal vehicle injection (Con) shows tight junction with typical hexagonal shape. **B.** RPE flat mount after subretinal OAβ_42_ injection (Subretinal OAβ_42_) shows intracellular Aβ and disrupted irregular expression of ZO-1. Orthogonal images indicates intracellular position of Aβ. **C.**, **D.** Optical zoom: ×2.4, a magnified portion of image a, b (enclosed in the white dotted box) to indicate intracellular Aβ and tight junction breakdown. **D.** Arrows indicate disrupted tight junctions. **E.** Representative images with 3-D reconstruction using Imaris software shows intracellular Aβ in RPE layer after subretinal injection of FITC-labeled OAβ_42._ Arrows indicate intracellular Aβ (green). Magnification, ×1000. Scale bar = 20 μm. Figures were selected as representative data from three independent experiments.

To evaluate cellular uptake of Aβ in RPE, we demonstrated intracellular Aβ uptake after subretinal injection of exogenous OAβ_42_. Seven days after subretinal OAβ_42_ injection, intracellular Aβ_42_ was found concomitantly with the breakdown of tight junction (Figure 1). While typical hexagonal pattern of ZO-1 was not disrupted in vehicle injected mice (Figure 1A), the disrupted and irregular pattern of ZO-1 was found in OAβ_42_ injected mice which indicated a breakdown of tight junction in RPE (Figure 1B). These specific changes of tight junction were shown in details (Figure 1C and 1D). We also confirmed the intracellular uptake of Aβ using FITC-labeled OAβ_42_ (Figure 1E). These data suggest that RPE uptakes exogenous Aβ, and this, in turn, leads to tight junction breakdown *in vivo*.

### Extracellular Aβ translocates into intracellular space via RAGE-mediated endocytosis in RPE

**Figure 2:**
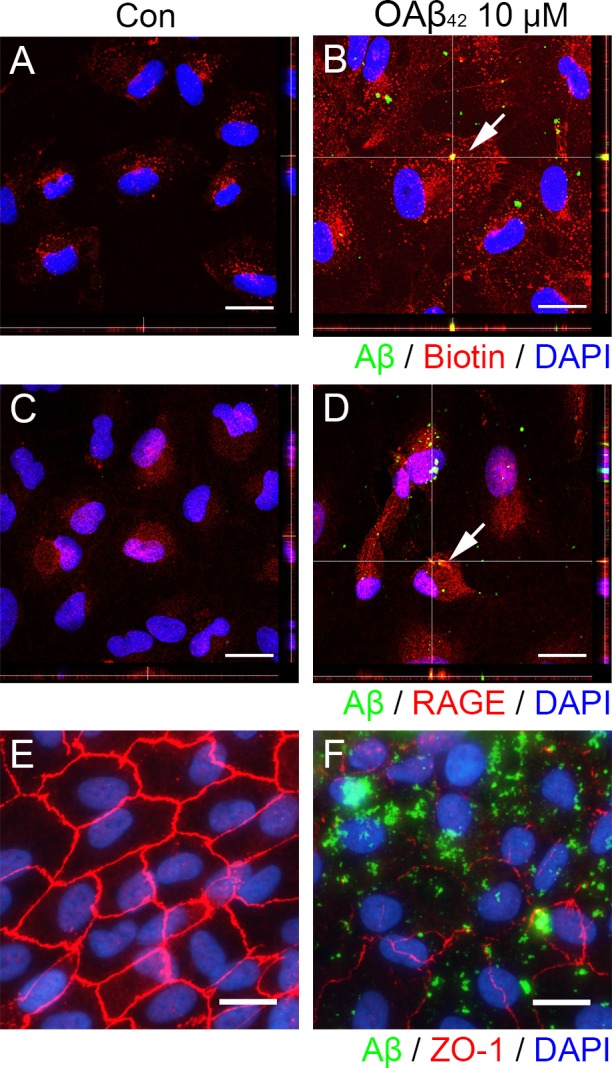
Extracellular Aβ translocates into intracellular space via RAGE-mediated endocytosis in RPE ARPE-19 cells were exposed to vehicle control **A.**, **C.** or OAβ_42_ 10 μM (B, D) for 60 min, fixed in 4% PFA, and stained by anti-human Aβ_42_ (green) and anti-biotin (red) or anti-RAGE (red). **A.** RPE cells shows basal level of endocytosis of cell membrane proteins (red). **B.** RPE cells treated with OAβ_42_ 10 μM shows increased level of endocytosis of cell membrane proteins (red) in orthogonal view with a 0.49 μm Z-step interval. A magnified portion of image B (enclosed in the white dotted box) indicates intracellular Aβ merged with biotinylated membrane proteins. Arrow indicates intracellular colocalization of Aβ and biotin. **C.** RPE cells shows RAGE expression. **D.** RPE cells treated with OAβ_42_ 10 μM shows internalized RAGE (red) in orthogonal view. A magnified portion of image D (enclosed in the white dotted box) indicates intracellular Aβ merged with internalized RAGE. Arrow indicates colocalization of Aβ and RAGE. **E.**, **F.** ARPE-19 cells were exposed to vehicle control **E.** or OAβ_42_ 10 μM **F.** for 24 h, fixed in 4% PFA, and stained by anti-human Aβ_42_ (green) and anti-ZO-1 (red). **E.** RPE cells show typical hexagonal shape tight junction. **F.** RPE cells show disintegrated and disorganized ZO-1 with intracellular Aβ. Magnification, ×1000. Scale bar = 20 μm. Figures were selected as representative data from three independent experiments.

In order to determine the mechanism how extracellular Aβ gets translocated into intracellular space in RPE, we demonstrated intracellular Aβ uptake with plasma membrane and RAGE after exogenous OAβ_42_ treatment. First, we assessed intracellular distribution of Aβ and biotinylated-cell surface protein after Aβ treatment. After biotinylation of cell surface protein, cells were incubated with vehicle or OAβ_42_ for 1 h, and were treated with 2-mercaptoethanesulfonate (MesNa) to remove any remaining biotin on the cell surface. While control cells exposed to vehicle alone showed some biotinylated-proteins in cytosol (Figure 2A), RPE cells exposed to OAβ_42_ showed significantly increased biotinylated-proteins in cytosol with intracellular Aβ (Figure 2B). Intriguingly, intracellular Aβ was overlapped with some internalized biotinylated-proteins (Figure 2B, inset) suggesting that Aβ could bind to cell surface protein and translocate into intracellular space together.

To access possible colocalization of RAGE and Aβ, cells were incubated with vehicle or OAβ_42_ for 1h, and the distribution of RAGE and Aβ was examined under confocal microscope (Figure 2C and 2D). Indeed, the colocalization of RAGE and Aβ was detected in RPE exposed to OAβ_42_ (Figure 2D). These data suggested that Aβ interacts with RAGE and is internalized into RPE.

To analyze the effect of intracellular Aβ on tight junction integrity in RPE, we studied tight junction of RPE cells exposed to vehicle or OAβ_42_ for 24 h (Figure 2E and 2F). Intracellular Aβ was concomitant with disrupted and disorganized ZO-1 expression (Figure 2F).

### siRNA-mediated knockdown of RAGE suppresses Aβ uptake in RPE

**Figure 3:**
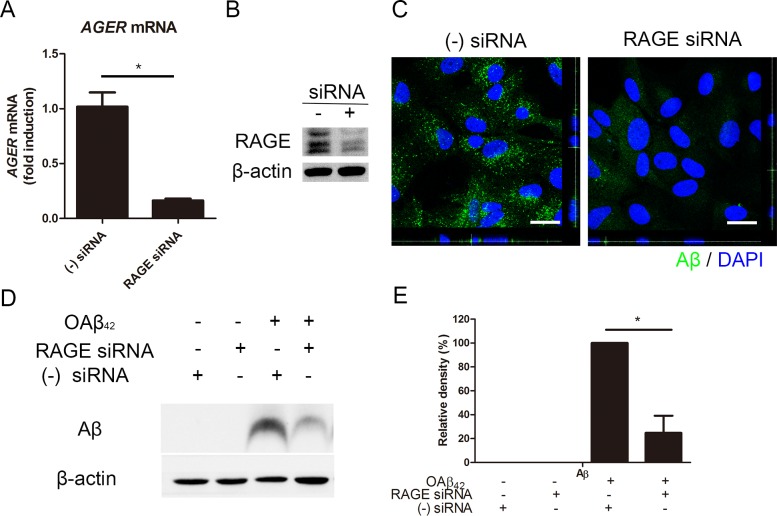
siRNA-mediated knockdown of RAGE suppresses Aβ uptake in RPE RAGE siRNA was transfected in ARPE-19 cells. Negative siRNA was used as a control. RPE cells were treated OAβ_42_ 10 μM for 24 h. Intracellular Aβ uptake is decreased in RPE with RAGE siRNA compared to RPE with negative siRNA. **A.** Relative expression of *AGER* mRNA is decreased in RPE cells with RAGE siRNA. **B.** RAGE expression is decreased in RPE cells with RAGE siRNA. **C.** Immunocytochemistry of Aβ_42_ (green) shows decreased intracellular Aβ in RPE cells with RAGE siRNA. **D.** Intracellular Aβ was evaluated by Western blot. **E.** Relative band density of Aβ was analyzed using ImageJ 1.42 software. β-actin was used as a loading control. Data are presented as mean ± SEM. in graphs. *p < 0.05 (two tailed, unpaired T-test). Figures were selected as representative data from three independent experiments.

To investigate the role of RAGE on Aβ uptake in RPE cells*,* we performed *in vitro* study in RPE cells treated with RAGE siRNA. siRNA-mediated knockdown effectively decreased *AGER* mRNA (0.16 ± 0.02 fold induction) compared to negative siRNA (1.00 ± 0.13, *p* < 0.05; Figure 3A). In accordance with this result, it effectively decreased RAGE expression in RPE (Figure 3B). Then, we examined intracellular Aβ uptake in RPE cells with RAGE siRNA. Interestingly, intracellular Aβ uptake was decreased in RPE with RAGE siRNA compared to RPE with negative siRNA under confocal microscope (Figure 3C). Consistent with these data, western blot results also showed that intracellular Aβ was significantly decreased in RPE with RAGE siRNA compared to RPE with negative siRNA (Figure 3D and 3E). These data showed that siRNA-mediated knockdown of RAGE suppressed Aβ uptake in RPE. This implied that RAGE played an important role in intracellular Aβ uptake in RPE.

### RAGE-mediated p38 MAPK signaling contributes to endocytosis of Aβ in RPE

**Figure 4:**
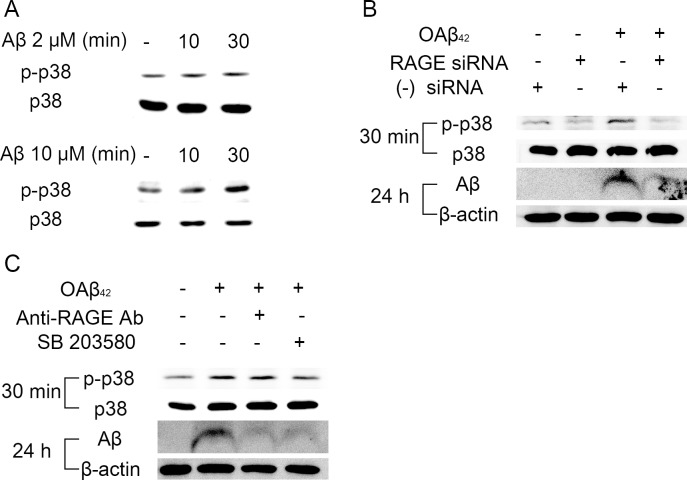
RAGE-mediated p38 MAPK signaling contributes to endocytosis of Aβ in RPE **A.** RPE cells were treated OAβ_42_ 2 μM or 10 μM for 30 min. Phosphorylation of p38 MAPK is increased at 30 min. **B.** RAGE siRNA was transfected in ARPE-19 cells. Negative siRNA was used as a control. RPE cells were treated OAβ_42_ 10 μM for indicated time (30 min and 24 h). Phosphorylation of p38 MAPK and intracellular Aβ uptake are decreased in RPE with RAGE siRNA compared to RPE with negative siRNA. **C.** RPE cells were pretreated with anti-RAGE neutralizing antibody (20 μg/ml, 2h) and SB 203580 (10 μM, 30 min) and were treated with OAβ_42_ 10 μM for indicated time (30 min and 24 h). Anti-RAGE neutralizing antibody and SB 203580 decrease intracellular Aβ in RPE. β-actin was used as an internal control. Figures were selected as representative data from three independent experiments.

RAGE is known to activate multiple downstream signaling pathways as a signal transduction receptor [18]. Based on the previous study [15], we demonstrated that Aβ-induced RAGE signaling activation might lead to Aβ uptake into RPE. We first examined the effect of Aβ treatment on phosphorylation of p38 MAPK. Treatment of OAβ_42_ for 30 min showed a dose-dependent increase in phosphorylated p38 MAPK although it did not affect total protein levels of p38 MAPK (Figure 4A).

Next, we studied whether Aβ-induced phosphorylation of p38 MAPK is mediated by RAGE. RAGE siRNA effectively suppressed Aβ-induced p38 MAPK phosphorylation, and reduced intracellular Aβ accumulation (Figure 4B). In addition, RPE cells pretreated with p38 MAPK inhibitor (SB 203580) showed strong inhibition of Aβ uptake, similar to RPE cells pretreated with anti-RAGE neutralizing antibody (Figure 4C). Thus, Aβ/RAGE-mediated p38 MAPK signaling contributes to intracellular Aβ uptake in RPE.

### Blockade of RAGE inhibits intracellular Aβ-induced tight junction breakdown in RPE

**Figure 5:**
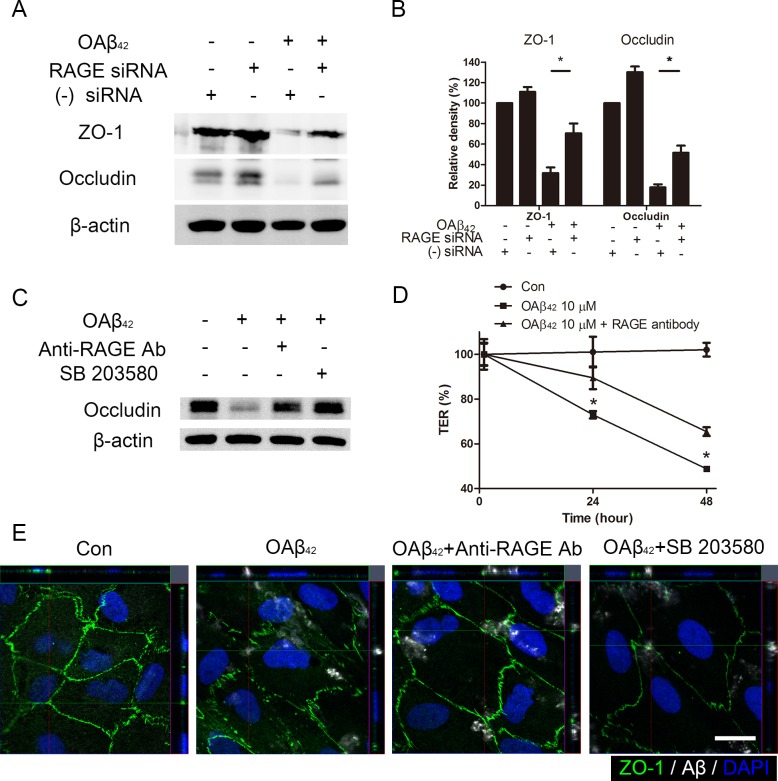
Blockade of RAGE inhibits intracellular Aβ-induced tight junction breakdown **A.** RAGE siRNA was transfected in ARPE-19 cells. Negative siRNA was used as a control. RPE cells were treated OAβ_42_ 10 μM for 24 h. Tight junction proteins (ZO-1 and occludin) were evaluated by Western blot. β-actin was used as an internal control. **B.** Relative band density was analyzed using ImageJ 1.42 software. **C.** RPE cells were pretreated with anti-RAGE neutralizing antibody (20 μg/ml, 2h) and SB 203580 (10 μM, 30 min) and were treated with OAβ_42_ 10 μM for 24 h. Occludin was evaluated by Western blot. β-actin was used as an internal control. **D.** Transepithelial electrical resistance (TER) was measured for 48 h and normalized to the TER value just after Aβ treatment. TER values with anti-RAGE neutralizing antibody (triangle) are compared to TER with OAβ_42_ treatment alone (square) at indicated time point. Data are presented as mean ± SEM. in graphs. *p < 0.05 (two tailed, unpaired T-test). **E.** RPE cells were pretreated with anti-RAGE neutralizing antibody (20 μg/ml, 2h) and SB 203580 (10 μM, 30 min), and were treated with OAβ_42_ 10 μM for 24 h. Cells were stained by anti-human Aβ_42_ (white) and anti-ZO-1 (green). Magnification, ×1000. Scale bar = 20 μm. Figures were selected as representative data from three independent experiments.

To determine whether inhibition of intracellular Aβ uptake could prevent Aβ-induced tight junction breakdown in RPE, we analyzed tight junction expression in RPE using western blot. We demonstrated that intracellular uptake of exogenous OAβ_42_ was decreased in RPE with siRNA-mediated knockdown of RAGE (Figure 4B). Indeed, siRNA-mediated knockdown of RAGE diminished the uptake of Aβ which subsequently inhibited intracellular Aβ-induced tight junction breakdown in RPE (Figure 5A and 5B). Consistent with these data, treatment of p38 MAPK inhibitor (SB 203580) and anti-RAGE neutralizing antibody also showed protective effect on Aβ-induced decrease of occludin expression in RPE (Figure 5C).

To determine the role of RAGE on barrier function of RPE after Aβ treatment, we measured transepithelial resistance (TER) in RPE for 48 h. The mean initial TER of controls was 46±4.5 ohms·cm2 when they reached a plateau. Anti-RAGE neutralizing antibody was sufficient to inhibit a decrease of TER during 48 h after OAβ_42_ treatment (*p* < 0.05; Figure 5D). In addition, tight junction breakdown with ZO-1 disruption after OAβ_42_ was also attenuated when ARPE-19 cells were treated with either p38 MAPK inhibitor or anti-RAGE neutralizing antibody (Figure 5E). These data suggested that intracellular Aβ uptake by RAGE contributed to breakdown of tight junction in RPE.

## DISCUSSION

In this study, we demonstrated that subretinal injection of Aβ led to intracellular uptake of Aβ and subsequent breakdown of tight junction in RPE *in vivo*. We also showed that intracellular Aβ contributed to breakdown of tight junction in RPE via the RAGE/p38 MAPK-mediated endocytosis. RAGE contributed to intracellular uptake of Aβ which resulted in the increase of tight junction breakdown in RPE. Our study of the mechanism of intracellular Aβ uptake in RPE might help to investigate the role of intracellular Aβ in the pathogenesis of dry AMD. Furthermore, we also suggested that the research field of Aβ should be expanded from AD to AMD, and from extracellular Aβ to intracellular Aβ.

A growing body of evidence suggests that intraneuronal Aβ contributes neuronal apoptosis in the early pathogenesis of AD [7, 8]. Intracellular Aβ_42_ is known to accumulate in 5XFAD mice brain prior to plaque formation [19]. We hypothesized that intracellular Aβ was also important in the early pathogenesis of AMD. However, the role of intracellular Aβ in RPE was only described in our previous study suggesting that intracellular Aβ in RPE could contribute to the development of AMD [9]. Aβ is predominantly secreted from the neuronal cells, but the mechanism of Aβ transport into the RPE remains to be fully elucidated in AMD.

Regarding to Aβ uptake by RPE cells, the mechanism of intracellular uptake of exogenous Aβ is not established. Although the main role of RPE is phagocytosis, RPE phagocytosis showed a remarkable specificity toward ROS via αvβ5 integrin [20, 21]. There were also several endocytotic pathways suggested as possible uptakes of Aβ in neurons and endothelial cells, including α7-NAch receptor [22], NMDA receptor [23], LDL receptor-related protein 1 [24], and RAGE [14]. Among these candidates, we demonstrated that RAGE contributed to intracellular Aβ uptake in RPE. RAGE is also important in retinal aging [25]. It is known that RAGE distributed at apical membrane of the RPE [11, 26]. In line with our results, Aβ/RAGE-mediated p38 MAPK signaling contributes to Aβ transport and neuronal dysfunction [15]. Activation of endocytosis is essential in regulating the p38 MAPK activity in endothelial cells [27]. Despite the evident role of RAGE in endocytosis of Aβ in this study, phagocytosis or other receptor mediated endocytosis could also contribute to intracellular uptake of Aβ. In addition, we also hypothesized that RAGE-mediated signaling itself (i.e. GSK-3β or NF-κB related signaling pathway) could affect tight junction or Aβ-related pathology in RPE. Thus, those Aβ-related mechanisms on RPE pathology should be investigated in further study.

The limitation of the study is that ARPE19 cells generate less effective tight junction than human fetal RPE (hfRPE) cells for *in vitro* study. It is also known that the staining with occludin in ARPE-19 cells is not good as that in hfRPE [28]. Thus, it is better to show the distribution of ZO-1 tight junction protein in immunocytochemistry for tight junction evaluation in ARPE-19 cells. Nonetheless, this study for the first time demonstrated that intracellular Aβ uptake was mediated by RAGE via the RAGE/p38 MAPK-mediated endocytosis in RPE.

In conclusion, intracellular Aβ uptake was mediated by RAGE in RPE. Intracellular Aβ contributed to breakdown of tight junction in RPE via the RAGE/p38 MAPK-mediated endocytosis. Blockade of RAGE with anti-RAGE antibody could block the intracellular accumulation of Aβ, and subsequently prevent the breakdown of tight junction in RPE. Thus, we suggest that blockade of RAGE can be a potential therapeutic target in AMD. Further study is warranted to develop new modality of RAGE-specific inhibitor for the treatment of AMD.

## MATERIALS AND METHODS

### Animals

All animal experiments in this study were in strict agreement with the Association for Research in Vision and Ophthalmology Statement for the Use of Animals in Ophthalmic and Vision Research and the guidelines of the Seoul National University Animal Care and Use Committee. Six-week-old, pathogen-free male C57BL/6J mice were purchased from Central Lab (Seoul, Korea).

### Reagents and antibodies

Rabbit anti-ZO-1 and anti-occludin antibodies, Lipofectamine RNAi max and other culture reagents were purchased from Life technologies (Gaithersburg, MD, USA). Anti-p38, anti-phospho p38, and anti-β-actin antibodies were purchased from Cell Signaling Technology Inc. (Beverly, MA, USA). Mouse anti-Aβ_42_ (12F4) antibody was purchased from Covance (Seoul, Korea). SB 203580 was purchased from EMD Millipore (Billerica, MA, USA). Anti-RAGE neutralizing mAb (MAB11451) and anti-RAGE antibody (AF1145) were purchased from R&D system Inc. (Minneapolis, MN, USA). EZ-Link Sulfo-NHS-SS-Biotin (Cat #21328) was purchased from Pierce Biotechnology Inc. (Rockford, IL, USA). RAGE siRNA and control negative siRNA were purchased from Bioneer (Daejeon, Korea). Aβ_42_ and FITC labeled Aβ_42_ were purchased from American peptide company Inc. (Sunnyvale, CA, USA).

### Cell cultures and siRNA transfection

ARPE-19 cells (American Type Culture Collection, Manassas, VA, USA) were used for human RPE cells. The cells were routinely maintained in DMEM/F12 containing 10% FBS, 100 U/mL penicillin, and 100 μg/mL streptomycin. RAGE siRNA and negative siRNA (50 nM) were transfected using Lipofectamine RNAi max in opti-MEM^®^ media according to the manufacturer's instructions. After reaching confluent, ARPE-19 cells were maintained in DMEM/F12 containing 1% FBS to make a polarization for the experiments [29].

### Preparation of oligomeric Aβ42 solution

OAβ_42_ solution were generated as the previously described method [9]. Aβ_42_ and FITC labeled Aβ_42_ (62-0-80B and 62-0-82B) were dissolved in hexafluoro-2-propanol (Sigma Aldrich, St. Louis, MO, USA) to a final concentration of 1 mg/ml at RT for 3 days. The peptide was aliquoted and dried under vacuum for 1 h. The aliquoted peptide was dissolved in DMSO to a final concentration of 2 mM. The protein concentration was measured using a BCA protein assay kit (Pierce Biotechnology Inc.). The Aβ_42_ stock in DMSO was diluted directly into DMEM/F12 at 10 μM, and incubated for 24 h at 4 ^°^C to make OAβ_42_.

### Subretinal injection

Twenty-week-old C57BL/6J mice were deeply anesthetized using a mixture of Zoletil 50^®^ (Virbac, Carros, France) and Rompun^®^ (Bayer Korea, Seoul, Korea) (3:1 ratio, 1ml/kg, i.p.). Then subretinal injection of 2 uL of 100 μM OAβ_42_ was performed using Nanofil syringe with 33 G blunt needle (World Precision Instruments Inc., Sarasota, FL, USA) under operating microscope (Leica Microsystems Ltd. Seoul, Korea). For the control mice, only 2 uL of DMEM/F12 media without Aβ was injected as a vehicle.

### Immunofluorescence staining

C57BL6/J mice (n = 12) were sacrificed 1 week after subretinal injection of OAβ_42_. After deep anesthesia, mice were sacrificed and globes were enucleated. Enucleated eyes were fixed in 4% paraformaldehyde for 24 h. For the flat mount of RPE/choroid complex, enucleated eyes were dissected out to remove neural retina, the RPE/choroid complex were gently flat mounted and fixed in methanol for 15 min at −20 ^°^C. After washing with PBS, RPE/choroid complex was incubated in Perm/Block solution (0.2% Triton-X 100 and 0.3% BSA in PBS) at RT for 1 h. Then, it was incubated overnight at 4 ^°^C with primary antibodies against rabbit anti-ZO-1 (1:100) and mouse anti-Aβ_42_ (1:100). After washing with PBS, it was incubated at RT for 1 h with secondary antibodies (Alexa Fluor 488 donkey anti-mouse IgG, 1:200 and Alexa Fluor 594 donkey anti-rabbit Ig G, 1:200). After washing with PBS, it was counterstained with 10 mg/ml DAPI (Sigma Aldrich). After washing with PBS, the RPE/choroid complex was mounted with Fluoromount™ Aqueous Mounting Medium (Sigma Aldrich) and observed under confocal microscope (Leica TCS STED, Leica Microsystems Ltd.).

### Biotinylation of membrane proteins

Biotinylation of cell membrane proteins was performed as previously described method [15]. ARPE-19 cells plated in 8-well culture slides were washed with PBS and incubated with PBS containing 300 μg/mL sulfo-NHS-SS-biotin at RT for 30 min. The biotinylation of membrane proteins was quenched using 1 M glycine in PBS at 4 ^°^C for 15 min. After additional two brief wash with 100 mM glycine in PBS to remove the residual biotin, cells were incubated with OAβ_42_ at 37^°^C for 1 h. Then, to terminate uptake of biotinylated proteins, cells were washed with cold NT buffer (150 mM NaCl, 1mM EDTA, 0.2% BSA, and 20 mM Tris, pH 8.6). Then, cells were incubated with 50 mM MesNa/NT buffer at 4 ^°^C for 30 min.

### Immunocytochemistry

ARPE-19 cells with confluence were incubated with OAβ_42_ (10 μM) in serum free DMEM/F12 medium for 24 h. After removal of the medium, cells were intensively washed with warm PBS and fixed with 4% paraformaldehyde at RT for 15 min. For the permeabilization, 0.2% Triton X-100 in PBS was treated for 10 min. After washing, the cells were incubated with 1% BSA in PBS at RT for 1 h. Cells were incubated overnight at 4 ^°^C with rabbit anti-ZO-1 (1:1000), mouse anti-Aβ (1:1000) and goat anti-RAGE (1:500). After washing, cells were incubated at RT for 1 h with secondary antibodies (Alexa Fluor 488, 594, 647 anti-mouse, rabbit, goat IgG, 1:500). Nucleus was counterstained with DAPI at RT for 10 min. After washing, the slides were mounted and observed under confocal microscope (Leica TCS STED).

### Western blotting

ARPE-19 cells were incubated with 10 μM OAβ_42_ for 30 min to detect p38 MAPK and for 24 h to detect intracellular Aβ and tight junction proteins. SB 203580 (10 μM, p38 MAPK inhibitor, EMD Millipore, 559395) was pretreated 30 min prior to OAβ_42_ treatment. Anti-RAGE neutralizing antibody (20 μg/ml) was pretreated 2 h prior to OAβ_42_ treatment. Cell proteins were extracted with RIPA buffer (Tris 50mM pH 7.4; NaCl 150 mM; SDS 0.1%; NaDeoxycholate 0.5%; Triton X-100 1%) with a complete protease inhibitor cocktail (Roche, Indianapolis, IN, USA). Twenty five micrograms of protein was separated by SDS-PAGE, and transferred to nitrocellulose membranes (GE healthcare life sciences, Piscataway, NJ, USA). After blocking in 5% BSA in PBST (0.1% Tween 20 in PBS), the membranes were incubated with primary antibodies for p38 (1:1,000), phospho-p38 (1:1,000), ZO-1 (1:1,000), occludin (1:2,000), Aβ_42_ (1:1000) and actin (1:5,000). The membranes were incubated with ECL substrate (DoGEN, Seoul, Korea) and exposed in ImageQuant™ LAS 4000 (GE healthcare life sciences). The band intensity analyzed using ImageJ 1.42 software (National Institutes of Health, Bethesda, MD, USA).

### Transepithelial electrical resistance measurement

The measurement of TER was performed by impedance analysis using EVOM2 TER (World Precision Instruments). Briefly, the cell covered transwell filters (0.4 μm, Corning Inc., NY, USA) coated with laminin were placed in this setup using 12-well plate. After stabilization of TER value on plateau, TER was measured with 10 μM OAβ_42_ and anti-RAGE neutralizing antibody (20 μg/ml) for 48 h. The TER value just after treatment was normalized to 100% for relative analysis.

### Real-time PCR

Total RNA was isolated from RPE cells using TRIzol reagent according to the manufacturer's instructions. cDNA was prepared with High Capacity RNA-to-cDNA kit. Real-time PCR was performed with StepOnePlus Real-time PCR System with TaqMan^®^ Fast Advanced Master Mix and specific Gene Expression Assays (*AGER* and *GAPDH*). All procedures were performed in accordance with the MIQE guidelines. All material and machine used in Real-time PCR was purchased from Life Technologies.

### Statistical analysis

Statistical analyses were performed using SPSS software version 18.0 (SPSS Inc., Chicago, IL, USA). Two-tailed unpaired T-test was used. *P* values less than 0.05 were considered to be statistically significant. Data and figures are depicted as mean ± SEM.
